# Retrospective Echocardiographic Analysis of West Syndrome During Adrenocorticotropic Hormone Therapy

**DOI:** 10.3389/fped.2022.889752

**Published:** 2022-05-10

**Authors:** Yoji Ikuta, Masaru Miura, Tomohide Goto, Sahoko Miyama

**Affiliations:** ^1^Higashi-Koganei Child Neurology and Epilepsy Clinic, Koganei, Japan; ^2^Department of Neurology, Tokyo Metropolitan Children’s Medical Center, Fuchu, Japan; ^3^Department of Cardiology, Tokyo Metropolitan Children’s Medical Center, Fuchu, Japan; ^4^Division of Pediatric Neurology, Kanagawa Children’s Medical Center, Yokohama, Japan

**Keywords:** West syndrome, treatment, ACTH, echocardiography, ventricular hypertrophy, diastolic dysfunction

## Abstract

**Background:**

Ventricular hypertrophy is a well-known side effect of adrenocorticotropic hormone (ACTH) therapy in patients with West syndrome (WS), but there are only a few reports of echocardiographic evaluation of these patients’ diastolic function.

**Methods:**

The present, retrospective study analyzed echocardiographic findings in 24 patients with WS treated with ACTH therapy between April 2010 and December 2014. The therapy protocol involved administering tetracosactide acetate 0.01–0.0125 mg/kg via intramuscular injection once a day for weeks 1–2, then gradually tapering off. Echocardiographic evaluation was done before treatment initiation and at weeks 1, 2, and 4 after the initiation of treatment.

**Results:**

The systolic and diastolic blood pressure values were elevated at week 1 after commencement of the therapy and remained elevated throughout its duration. Both the interventricular septal end-diastolic thickness and left ventricular posterior wall end-diastolic diameter increased in thickness at week 1 and remained thickened. None of the patients experienced heart failure or systolic dysfunction. Early diastolic mitral flow velocity (E)/early diastolic mitral annular velocity (E′) increased at week 1 and remained high at weeks 2 and 4. The E wave deceleration time (DcT) was prolonged at week 2 and returned to the baseline at week 4.

**Conclusion:**

Increased ventricular wall thickness, decreased diastolic capacity, and elevated BP were noted in children with WS during ACTH therapy. Cardiac function, including ventricular wall thickness and diastolic function, should be monitored during ACTH therapy. E/E′ and DcT are useful in evaluating diastolic function.

## Introduction

West syndrome (WS), a developmental and epileptic encephalopathy of infancy, was first described in 1841 by West (1). Patients with WS typically present with epileptic spasms, psychomotor developmental arrest, and a characteristic electroencephalographic (EEG) pattern known as hypsarrhythmia. The major options for treatment of WS are adrenocorticotropic hormone (ACTH) therapy and antiepileptic drugs, mainly vigabatrin (2). Vigabatrin can have the severe adverse effect of irreversible tunnel vision and in Japan can be prescribed only at registered facilities. Because ACTH therapy has been used widely for a longer time and its efficacy is well-established, it is preferred to vigabatrin at many treatment facilities (3).

On the other hand, ACTH therapy can also have various, significant adverse effects (4), among which ventricular hypertrophy occurs frequently (5, 6); thus, many ACTH therapy protocols recommend regular echocardiographic evaluation. Nonetheless, only a few, previous studies have evaluated echocardiographic findings during ACTH therapy, in particular its effect on diastolic function. Kosuda et al. showed a trend toward decreasing early-to-late ventricular filling ratio (E/A) after commencement of ACTH therapy, but no statistically significant change was found in the diastolic capacity (7). Kutluk et al. reported that ACTH treatment had no significant adverse effect on cardiac function (8). However, these reports examined a small number of cases, and cardiac diastolic function was not well investigated.

At the study center, echocardiography screening tests are routinely incorporated into the ACTH therapy protocol for patients with WS. We therefore examined possible indices of cardiac function, especially the diastolic function, during ACTH treatment.

## Methods

### Study Design

We retrospectively investigated patients with a diagnosis of West syndrome who received ACTH therapy after admission to the Department of Neurology at Tokyo Metropolitan Children’s Medical Center between April 2010 and December 2014. Twenty-six patients were identified as candidates for our study. Of these, 24 were analyzed after excluding two patients with an underlying disease which might have influenced cardiac function; one patient had Williams syndrome with supra-aortic stenosis, and the other one had Leigh’s encephalopathy.

During hospitalization for ACTH treatment, vital signs, including blood pressure (BP), were assessed three times a day. The BP values obtained in the morning when the ACTH injection was given were analyzed. Echocardiographic evaluation was performed before the start of therapy and at the end of weeks 1, 2, and 4 after the commencement of treatment. The systolic functions of ejection fraction (EF) and percent fractional shortening (%FS); the diastolic functions of left ventricular early diastolic inflow velocity (E)/late diastolic mitral flow due to atrial contraction (A), E/early diastolic mitral annual velocity (E′), and E wave deceleration time (DcT); and cardiac wall thickness in terms of the interventricular septal end-diastolic thickness (IVSd) and left ventricular posterior wall end-diastolic diameter (LVPWd) were assessed.

### Adrenocorticotropic Hormone Therapy Protocol

In the standard dosage group, tetracosactide acetate 0.01–0.0125 mg/kg was administered via intramuscular injection once daily during weeks 1–2, every 2 days during week 3, and once every 3 days during week 4. Patients with no improvement in seizure frequency or EEG findings at week 1 or 2 after the commencement of therapy received an increased dosage and/or extended daily administration period.

### Statistical Analysis

BP and echocardiographic data during ACTH therapy were analyzed and expressed as the mean and standard error. Assuming that the values were normally distributed, a paired sample *t*-test was used to compare the changes with the values obtained before the start of treatment. The *p*-values were two-tailed, with *p* < 0.05 indicating statistical significance. All statistical analyses were performed using STATA/IC 15 (Stata Corp., LLC, College Station, TX, United States).

### Ethical Matters

The study complied with the ethical guidelines of the Declaration of Helsinki and those of the Ministry of Health, Labour and Welfare of Japan for medical and biological research involving human subjects. This study was approved by the Ethics Review Board of Tokyo Metropolitan Children’s Medical Center (ID: 2121b-57). Informed consent was implied by an opt-out clause as described on the institutional website because only deidentified information was collected from medical records.

## Results

Twenty-four patients were enrolled, and ACTH therapy was started at ages 2–19 months (median: 7 months). The sex ratio was almost equal, with 11 male and 13 female patients. Symptomatic patients accounted for 79% of the total; five had hypoxic-ischemic encephalopathy, three had psychomotor developmental delay, two had cerebral infarction, and the remainder had brain malformation, Down syndrome, other chromosomal abnormalities or neonatal subdural hemorrhage. EEG improved in 23 (96%) of all the treated patients, but seven (29%) needed enhanced treatment, such as an increased ACTH dosage and/or extended daily administration period, due to poor improvement in their EEG findings or seizure occurrence. Among these patients, five (21%) had recurrent cases. Exception for one patient (4%) with a subdural hematoma, none experienced any critical adverse effects of ACTH therapy, such as gastrointestinal hemorrhage or hydrocephalus.

**FIGURE 1:**
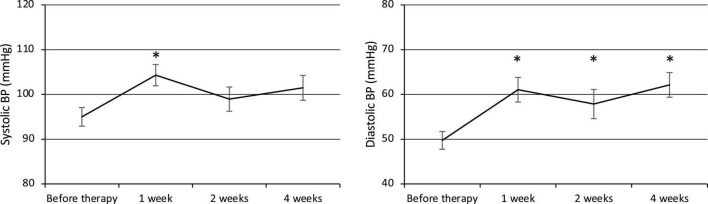
Changes in systolic and diastolic BP. BP, Blood pressure. *p < 0.05 for comparisons with pretherapy values. The line graph connects the mean values, and the error bars show the standard error ranges. Systolic and diastolic blood pressure were elevated after 1 week.

Both the systolic and diastolic BP values were elevated at week 1 after the commencement of therapy (Figure 1). The systolic BP was 95.0 ± 2.1 mmHg before therapy (*n* = 23) and increased significantly to 104.3 ± 2.4 mmHg at week 1 (*p* = 0.007, *n* = 23), then decreased to 99.0 ± 2.7 mmHg at week 2 (*n* = 21) and 101.5 ± 2.8 mmHg at week 4 (*n* = 23). The diastolic BP also increased significantly from 49.7 ± 2.0 mmHg before therapy (*n* = 23) to 61.0 ± 2.8 mmHg at week 1 (*p* = 0.001, *n* = 23) and remained elevated at 57.9 ± 3.3 mmHg at week 2 (*p* = 0.03, *n* = 21) and 62.1 ± 2.7 mmHg at week 4 (*p* = 0.0002, *n* = 23).

**FIGURE 2:**
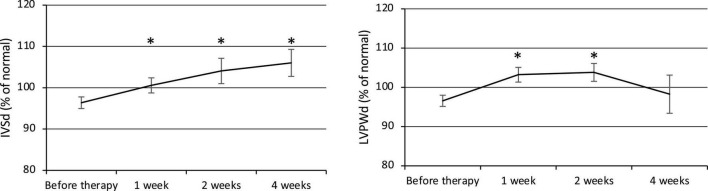
Changes in ventricular wall thickness (IVSd and LVPWd). IVSd, Interventricular septal end-diastolic thickness. LVPWd, Left ventricular posterior wall end-diastolic diameter. *p < 0.05 for comparisons with pretherapy values. The line graph connects the mean values, and the error bars show the standard error ranges. Both the IVSd and LVPWd increased in thickness after 1 week.

Both the IVSd and LVPWd of the ventricular wall increased at week 1 after the commencement of therapy (Figure 2). The IVSd (% of normal) was 96.4 ± 1.4% before therapy (*n* = 23) and gradually increased to 100.6 ± 1.8% at week 1 (*p* = 0.02, *n* = 23), 104.1 ± 3.1% at week 2 (*p* = 0.01, *n* = 22), and 106.0 ± 3.3% at week 4 (*p* = 0.003, *n* = 22). The LVPWd (% of normal) was 96.6 ± 1.4% before therapy (*n* = 23) and significantly increased to 103.2 ± 1.9% at week 1 (*p* = 0.0004, *n* = 23) and 103.8 ± 2.3% at week 2 (*p* = 0.005, *n* = 22), then decreased to 98.3 ± 4.9% at week 4 (*n* = 22). BP was elevated along with the ventricular wall thickening, but no statistically significant relationship was observed.

The EF was 75.8 ± 0.9 before therapy (*n* = 24) and remained at 78.9 ± 1.1 at week 1 (*n* = 24), 78.9 ± 1.0 at week 2 (*n* = 22), and 76.2 ± 1.0 at week 4 (*n* = 23). The% FS was 38.0 ± 0.9% before therapy (*n* = 24) and remained at 41.3 ± 1.0% at week 1 (*n* = 24), 40.8 ± 1.0% at week 2 (*n* = 22), and 38.3 ± 0.9% at week 4 (*n* = 23). The EF and% FS did not decrease during therapy, and none of the patients experienced heart failure or systolic dysfunction.

**FIGURE 3:**
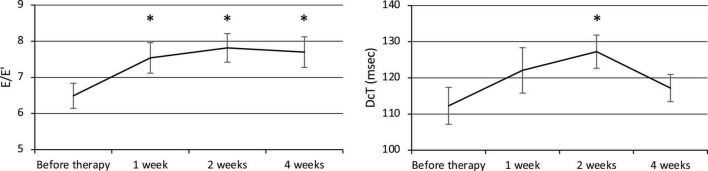
Changes in diastolic function (E/E′ and DcT). E, Left ventricular early diastolic inflow velocity. E′, Early diastolic mitral annual velocity. A, Late diastolic mitral flow due to atrial contraction. DcT, Deceleration time of E wave. *p < 0.05 for comparisons with pretherapy values. The line graph connects the mean values, and the error bars show the standard error ranges. The E/E′ increased after 1 week, and the DcT was prolonged after 2 weeks.

The E/E′ increased at weeks 1, 2, and 4, and the DcT was prolonged at week 2 after the commencement of therapy (Figure 3), but the E/A did not change significantly. The E/E′ was 6.49 ± 0.35 before therapy (*n* = 24) and significantly increased to 7.54 ± 0.42 at week 1 (*p* = 0.007, *n* = 24), 7.82 ± 0.39 at week 2 (*p* = 0.0008, *n* = 22), and 7.70 ± 0.42 at week 4 (*p* = 0.005, *n* = 24). The E/A was 1.29 ± 0.05 before therapy (*n* = 17), 1.38 ± 0.07 at week 1 (*n* = 17), 1.37 ± 0.09 at week 2 (*n* = 16), and 1.33 ± 0.05 at week 4 (*n* = 17). The DcT was 112.3 ± 5.1 ms before therapy (*n* = 21), 122.1 ± 6.3 ms at week 1 (*n* = 21), increased to 127.3 ± 4.6 ms at week 2 (*p* = 0.02, *n* = 21), then decreased to 117.2 ± 3.8 ms at week 4 (*n* = 22).

## Discussion

Few reports have examined cardiac function, especially diastolic cardiac function, in children with West syndrome during ACTH therapy, in detail. The present study found increased ventricular wall thickness, decreased diastolic function, and elevated BP during therapy, suggesting that not only ventricular wall thickness but also diastolic function, such as the E/E′ and DcT, should be monitored by echocardiography.

In the present study, the diastolic function changed significantly during therapy although none of the patients experienced heart failure or decreased systolic function. One report demonstrated a trend toward decreased E/A after the commencement of ACTH therapy but found no statistically significant change in diastolic capacity (7). In the present study, the E/E′ and DcT, but not the E/A, showed transient but statistically significant diastolic dysfunction; both parameters peaked at weeks 1–2, then normalized by week 4. Thus, the E/E′ and DcT should be included in an assessment of diastolic function during ACTH therapy.

Both the IVSd and LVPWd thickened markedly at week 1; then, the LVPWd leveled off, but the IVSd tended to increase during therapy. Previous studies demonstrated echocardiographically that the time to the onset of hypertrophic cardiomyopathy was 8 weeks (9) while the left ventricular free wall and septal wall dimensions increased from 13 to 46 days after the commencement of therapy (5) and that significant changes occurred earlier than in the present study. However, these reports assessed the changes at a minimum of 13 days after the commencement of therapy. Since the present study assessed cardiac function earlier and more frequently than these reports, we assumed that the myocardial changes occurred earlier. In addition, BP was elevated along with the ventricular wall thickening, but no significant association was found between the two events.

Ventricular wall thickening during ACTH therapy occurs as a result of various, biological effects of ACTH. Edema or deposition of glycogen in the myocardial tissue and hyperinsulinism are other, potential causes (6). Basic research has demonstrated that ACTH hypertension is related to the inhibition of α2-Na^+^ -K^+^ -ATPase on vascular smooth muscle by endogenous cardiotonic steroids (10). Therefore, myocardial thickening and elevated BP may occur independently (5). Since both myocardial hypertrophy and hypertension are common side effects of ACTH therapy, it is possible that they influence each other. Moreover, an animal study has shown that ACTH activated the local corticosterone synthesis pathway in the heart (11), possibly inducing hypertrophy and degeneration of myocytes (12) and leading to decreased cardiac wall flexibility and diastolic dysfunction.

The present study was retrospective and monocentric; it therefore has a number of limitations. The study pool consisted of only 24 patients, of whom five had recurrent West syndrome. Thus, the statistical power was inadequate. Further, only four patients had follow-up echocardiography after treatment completion; hence, we were unable to confirm whether the cardiac function parameters returned to baseline. Nonetheless, there were no clinical problems with cardiac function during or after treatment in any of the patients. Another consideration is that time has passed since the data collection period, and trends in West syndrome treatment have changed. At the time of the study, vigabatrin had not yet been approved for the treatment of West syndrome in Japan. In recent years, oral steroids and vigabatrin have become widely known as treatments with an efficacy equivalent to that of ACTH (2). However, ACTH is still the first-line treatment in Japan (3), and it would be meaningful to examine the follow-up of adverse events during ACTH therapy.

## Conclusion

The present study demonstrated abnormal changes not only in ventricular wall thickness but also in the diastolic function in children with West syndrome during ACTH therapy. The E/E′ and DcT should be monitored by echocardiography in patients with West syndrome to evaluate diastolic dysfunction.

## Data Availability Statement

The raw data supporting the conclusions of this article will be made available by the authors, without undue reservation.

## Ethics Statement

The studies involving human participants were reviewed and approved by the Ethics Board of Tokyo Metropolitan Children’s Medical Center. Written informed consent to participate in this study was provided by the participants’ legal guardian/next of kin.

## Author Contributions

YI, MM, TG, and SM designed the study. YI undertook data collection, data analysis, and drafting the manuscript. All authors critically reviewed the manuscript, approved the version herewith submitted, agreed to be accountable for all aspects of the work.

## Conflict of Interest

The authors declare that the research was conducted in the absence of any commercial or financial relationships that could be construed as a potential conflict of interest.

## Publisher’s Note

All claims expressed in this article are solely those of the authors and do not necessarily represent those of their affiliated organizations, or those of the publisher, the editors and the reviewers. Any product that may be evaluated in this article, or claim that may be made by its manufacturer, is not guaranteed or endorsed by the publisher.
